# Interactions of newly synthesized platinum nanoparticles with ICR-191 and their potential application

**DOI:** 10.1038/s41598-019-41092-6

**Published:** 2019-03-21

**Authors:** Agnieszka Borowik, Rafal Banasiuk, Natalia Derewonko, Michal Rychlowski, Marta Krychowiak-Masnicka, Dariusz Wyrzykowski, Magdalena Ziabka, Anna Woziwodzka, Aleksandra Krolicka, Jacek Piosik

**Affiliations:** 1 University of Gdansk, Intercollegiate Faculty of Biotechnology UG and MUG, Laboratory of Biophysics, Abrahama 58, Gdansk, 80-307 Poland; 2 University of Gdansk, Intercollegiate Faculty of Biotechnology UG and MUG, Laboratory of Biologically Active Compounds, Abrahama 58, Gdansk, 80-307 Poland; 3 University of Gdansk, Intercollegiate Faculty of Biotechnology UG and MUG, Laboratory of Virus Molecular Biology, Abrahama 58, Gdansk, 80-307 Poland; 4 University of Gdansk, Faculty of Chemistry, Wita Stwosza 63, Gdansk, 80-308 Poland; 5 AGH University of Science and Technology, Faculty of Materials Science and Ceramics, Department of Ceramics and Refractories, Krakow, 30-059 Poland

## Abstract

One of the greatest challenges of modern medicine is to find cheaper and easier ways to produce transporters for biologically active substances, which will provide selective and efficient drug delivery to the target cells, while causing low toxicity towards healthy cells. Currently, metal-based nanoparticles are considered a successful and viable solution to this problem. In this work, we propose the use of novel synthesis method of platinum nanoparticles (PtNPs) connected with their precise biophysical characterization and assessment of their potential toxicity. To work as an efficient nanodelivery platform, nanoparticles should interact with the desired active compounds spontaneously and non-covalently. We investigated possible direct interactions of PtNPs with ICR-191, a model acridine mutagen with well-established biophysical properties and mutagenic activity, by Dynamic Light Scattering, fluorescence spectroscopy, and Isothermal Titration Calorimetry. Moreover, to determine the biological activity of ICR-191-PtNPs aggregates, we employed Ames mutagenicity test, eukaryotic cell line analysis and toxicity test against the model organism *Caenorhabditis elegans*. PtNPs’ interesting physicochemical properties associated to the lack of toxicity in a tested range of concentrations, as well as their ability to modulate ICR-191 biological activity, suggest that these particles successfully work as potential delivery platforms for different biologically active substances.

## Introduction

For many years, platinum compounds have been broadly used as antineoplastic agents in diverse types of cancer treatment therapies (mostly as Pt^II^ coordination complexes, such as cisplatin, carboplatin, oxaliplatin, nedaplatin, lobaplatin, and heptaplatin). Briefly, their mechanism of action relays on platinum ions forming bonds with DNA bases, which causes DNA helix damage, arrest of the cell cycle and consequent apoptotic death. Platinum-containing chemotherapeutic drugs are efficient, but their usage is associated with serious dose-dependent adverse effects and increasing drug resistance^1–4^. The alarming fact is that many types of cancer cells demonstrate natural resistance to platinum-based drugs, which can further increase after the exposure to chemotherapy treatments^1,4–9^. Due to what, there is an urgent need to create new strategies overcoming drugs resistance in cancer cells. One of the currently discussed solutions is the development of platinum polyprodrug delivery^1,9^. According to the authors’ conclusions, such combination of the drug with nanoplatforms can provide enhanced cancer selectivity, reduction of the drug-related adverse effects, as well as protection of carried molecules from metabolic degradation. After entering the cell, cisplatin prodrugs might be released from the transporting complex due to degradation of cisplatin polyprodrug nanoplatforms by cathepsin B, an enzyme which is known to be overexpressed in diverse cancer cells.

On the other hand, nanoparticles attract much attention due to their unique properties and possible application in many scientific areas. One of the extensively studied examples of metal-based nanostructures are platinum nanoparticles at the platinum oxidation state 0 (Pt°, PtNPs). They are characterized by small size, high surface to mass ratio, high reactivity and electrocatalytic properties^2,3,10^. Therefore, they might serve as an efficient catalyst for many important chemical reactions, as well as common conductive materials in electronics and optics. PtNPs usage is not only limited to its physicochemical applications, but they can also play a role in nanodelivery devices, or can be utilized as co-treating factors in medicine, a new promising strategy of treatment. The probable development of PtNPs-based platforms for drugs transporting might provide several benefits such as enhanced cellular uptake, precise drug delivery, improved anticancer efficiency with lower toxicity, controlled biodistribution, accumulation at the tumor site, as well as reduced resistance^2,11^. Additionally, expected controlled transport and higher selectivity might reduce systemic drug exposure^3,6,11–13^. Several reports suggest that the use of nanoparticles as a component of synergistic therapies in cancer treatment not only enables cellular targeting, but also contributes to the reduction of adverse effects risk, increase in therapeutic efficiency and improvement of long-term prognosis for patients^6–8,13^.

PtNPs behave differently than platinum-based compounds, but they possess similar efficient anticancer activity^2,3,5,14,15^. After entering the cell through passive diffusion, PtNPs exert size, concentration and time-dependent toxicity, caused by the introduction of strand breaks in the DNA. It leads to the inhibition of replication, cell growth arrest, and apoptosis. Another possible mechanism of PtNPs action involves inhibition of metabolic activity of the cells, generation of hydroxyl radicals and release of active Pt^2+^ ions, strategy which is currently used in radiotherapy^5,14–18^. At certain concentration, PtNPs might also act as antioxidants^19^. In addition, some scientific reports hypothesize the potential use of PtNPs as coatings for sensors detecting glucose or other biomolecules^10^.

Nanoparticles designed for therapeutic usage require a uniform dispersion, low aggregation, non-toxicity towards healthy cells and the ability to interact with co-transported molecules. Unfortunately, the PtNPs synthesis techniques described in the literature so far are time and cost consuming, involving innumerous reaction steps and bulky equipment^3,10,16,18,20^.

In the present work, we propose a novel PtNPs synthesis method, which is inexpensive and efficient, simultaneously allowing for the precise control of nanostructure dimensions and shape. Furthermore, to assess the potential of these synthetic PtNPs as delivery platforms for biologically active agents, we applied complex biophysical and biological analyses. As a model of biologically active compound, we investigated the well-known and widely described acridine mutagen ICR-191. We evaluated whether ICR-191 molecules are able interact directly with PtNPs, in a non-covalent manner. In addition, we tested whether such interactions may influence the biological effects caused by the compound on bacteria, neoplastic and non-neoplastic eukaryotic cell lines. Moreover, we used *Caenorhabditis elegans* model to test the toxicity of PtNPs towards living multicellular organisms.

## Results and Discussion

**Figure 1:**
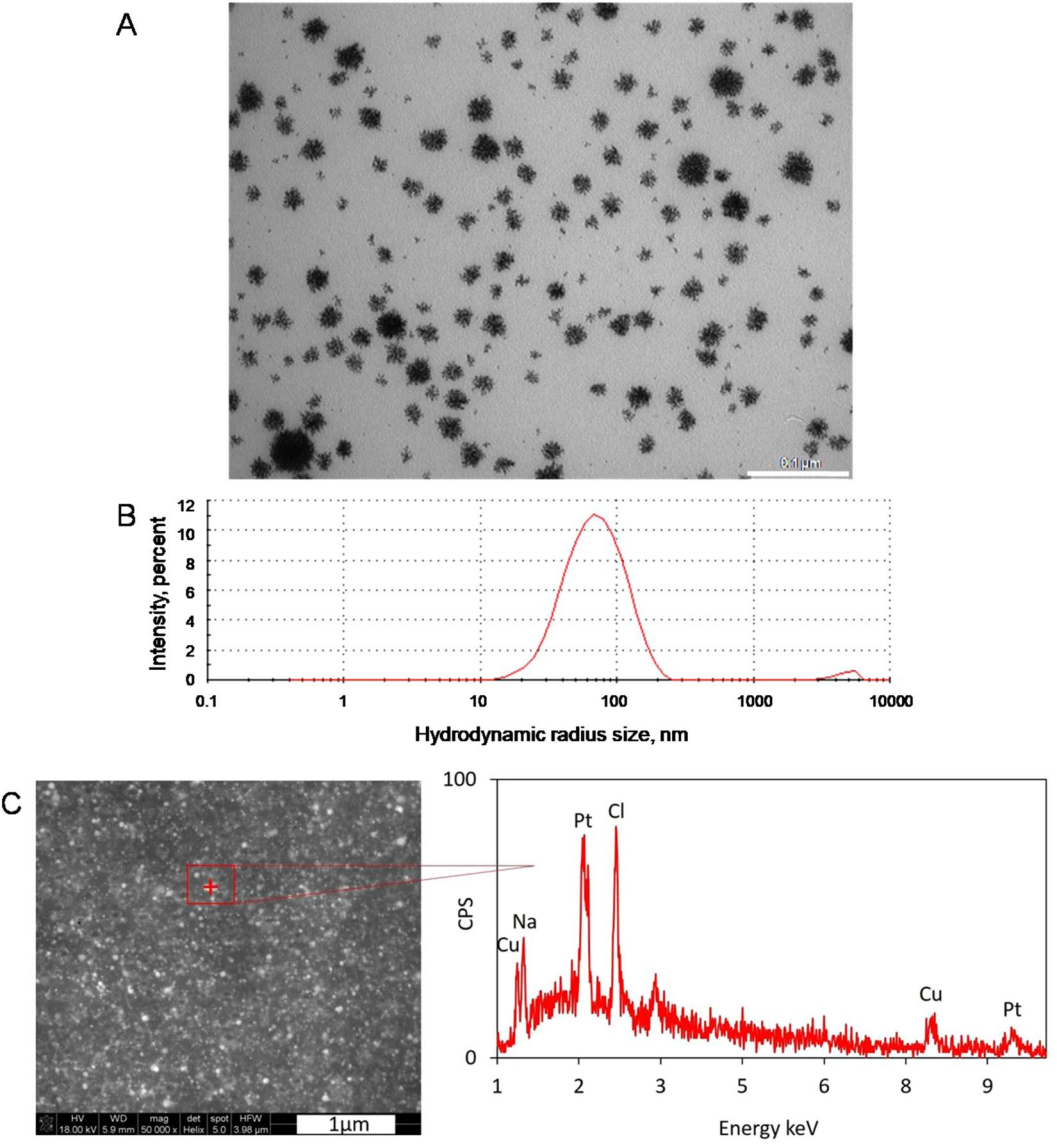
Platinum nanostructures obtained during novel synthesis: (**A**) platinum nanoparticles observed under TEM microscopy and (**B**) overall average size of platinum nanoparticles (concentration: 0.16 µg/mL) equals to 62.49 nm determined by DLS measurements and (**C**) SEM and EDS spectrum of platinum nanoparticles measured at the point marked with a red cross.

Ideally, an efficient approach to synthesize platinum nanostructures should be simple and free of costly reagents. The novel synthesis method proposed here differs from the already existing methods, as it is a less time and money consuming procedure, as it uses reagents found to be less aggressive for the environment. Depending on the process’ parameters, it was possible to establish the size of round-shaped platinum nanostructures between 10 to 80 nm, remembering that a single sphere can be as small as 1 nm. These details could be observed by transmission electron microscopy images (Fig. 1A). In addition, the hydrodynamic radii size of newly synthesized nanostructures was controlled by DLS analysis. The Z-average value measured for PtNPs mixture, an overall average size of aggregates in a tested solution, reaches 62.49 nm when the concentration of nanoparticles was equal to 0.16 µg/mL (Fig. 1B). The size of the PtNPs synthesized is similar to those obtained by other research groups^5,14,16,20,21^. It is worth to underline that Porcelet *et al*.^5^, Gehrkeet *et al*.^14^ and Mohammadi *et al*^16^. unanimously suggest the existence of an inversely proportional relationship between sizes of the nanoparticles, and their bioavailability for the cells. In Fig. 1C SEM image and EDS spectrum of PtNPs are presented. Based on the qualitative chemical analysis, platinum (Pt), copper (Cu) and additionally sodium (Na) and chlorine (Cl) were identified. The presence of copper is related to the synthesis process itself, which is carried out in the flow reactor made of copper capillary. Sodium and chlorine elements come from a water bath. However, after performing the quantitative analysis measurements with correction of sodium and chlorine we proved that the total amount of platinum is estimated about 86–90 percent by weight and copper is 10–14 percent by weight.

The consideration of PtNPs as potential nanodelivery devices should be preceded by a thorough understanding of their nanoformulation mechanism with drugs. The idea of using nanoparticles as transporters for different molecules, basing on the mixed aggregates formation as a result of non-covalent direct interactions between them, is not new. Nanostructures, such as PtNPs, can and sequestrate molecules in temporary heteroaggregates and in this way penetrate through cells membrane. Many authors indicate that this phenomenon might be the effect of electrostatic interactions, van der Waals forces, as well as hydrogen bond formation. Unfortunately, precise mechanism of action is still unexplained^6,22,23^. To address the ability of PtNPs to interact directly with aromatic ICR-191 molecules and to form mixed aggregates, we conducted UV-vis spectroscopy analysis. We performed two independent experiments: in the first one, ICR-191 solution was titrated with PtNPs; in the second PtNPs was titrated with increasing amounts of acridine mutagen. Unfortunately, light dispersion caused by PtNPs particles blocked proper biophysical analysis of registered spectra, and allowed us to only perform qualitative assumptions Figs S1 and S2 (Supporting Information). Nevertheless, visible changes in spectra might indicate that PtNPs-ICR-191 heteroaggregates are spontaneously formed. Similar optical changes were observed in a previous analysis done by our research group, where carbon nanoparticles fullerene C_60_ were also titrated with ICR-191^22^.

**Figure 2:**
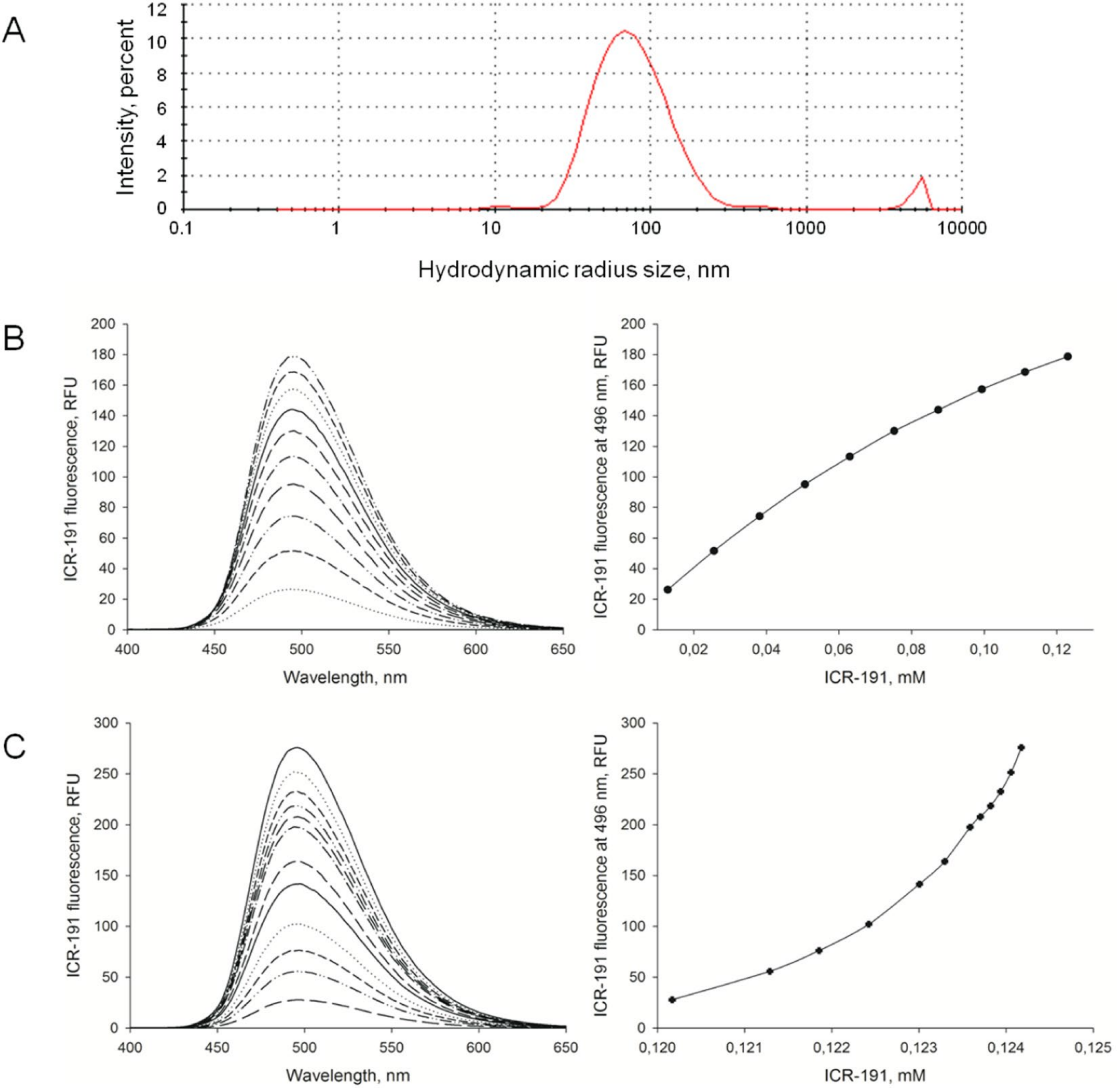
Aggregation of platinum nanoparticles with ICR-191: (**A**) overall average size of aggregates containing PtNPs and ICR-191 (concentrations respectively: 0.15 µg/mL and 0.12 mM) equals to 75.07 nm and (**B**) ICR-191 fluorescence emission spectrum (Ex = 340 nm, Em = 400–650 nm) measured for PtNPs (primary concentration: 32 µg/mL) titrated with increasing amount of ICR-191 (concentration range: 13–123 µM). Also shown as relationship between the fluorescence intensity at the maximum point (496 nm) and the ICR-191 concentration, and (**C**) ICR-191 fluorescence emission spectrum (Ex = 340 nm, Em = 400–650 nm) measured for ICR-191 (primary concentration: 124 µM) titrated with increasing amount of PtNPs (concentration range: 3.2–32 µg/mL). Also shown as relationship between the fluorescence intensity at the maximum point (496 nm) and the ICR-191 concentration.

To verify direct interactions, further experimental approaches were applied. In DLS analysis, the Z-average value determined for mixture containing both PtNPs and ICR-191 at concentrations of 0.15 µg/mL and 0.12 mM, respectively, was equal to 75.07 nm (Fig. 2A). There was a visible increase in the hydrodynamic radius size, which was likely caused by the nanoformulation of PtNPs-ICR-191 heteroaggregates. Similar extensions of the hydrodynamic radius were previously observed in analysis evaluating the heteroaggregation of fullerene C60 with ICR-191^22^, and with the anticancer drugs doxorubicin^24^ and cisplatin^25^.

ICR-191 has the ability to fluoresce, so we used it for further research assessing its potential nanoformulation with PtNPs by measuring the changes in the intensity of fluorescence light. In the first stage of the experiment acridine mutagen emission spectrum was registered with the excitation wavelength = 340 nm and emission wavelengths = 400–650 nm Fig. S3 (Supporting Information). Our synthesized PtNPs do not exert fluorescence themselves (data not shown). However, nanoparticles produced by other scientists may have such capabilities^26,27^. Figure 2B shows spectra of PtNPs (primary concentration: 32 µg/mL) titrated with increasing amounts of ICR-191 (concentration range: 13–123 µM), while Fig. 2C presents spectra of ICR-191 (primary concentration: 124 µM) titrated with increasing amount of PtNPs (concentration range: 3.2–32 µg/mL). The visible increase in fluorescence intensity at maximum point (496 nm) in Panel B, as well as the decrease in Panel C allow supposing that PtNPs interact with ICR-191 molecules, affecting its light emission properties. Notwithstanding, there is a possibility that ICR-191 concentrations changes and PtNPs light scattering might influence the obtained data. To exclude this risk, we performed additional thermodynamical analysis, thus removing potential doubts.

**Figure 3:**
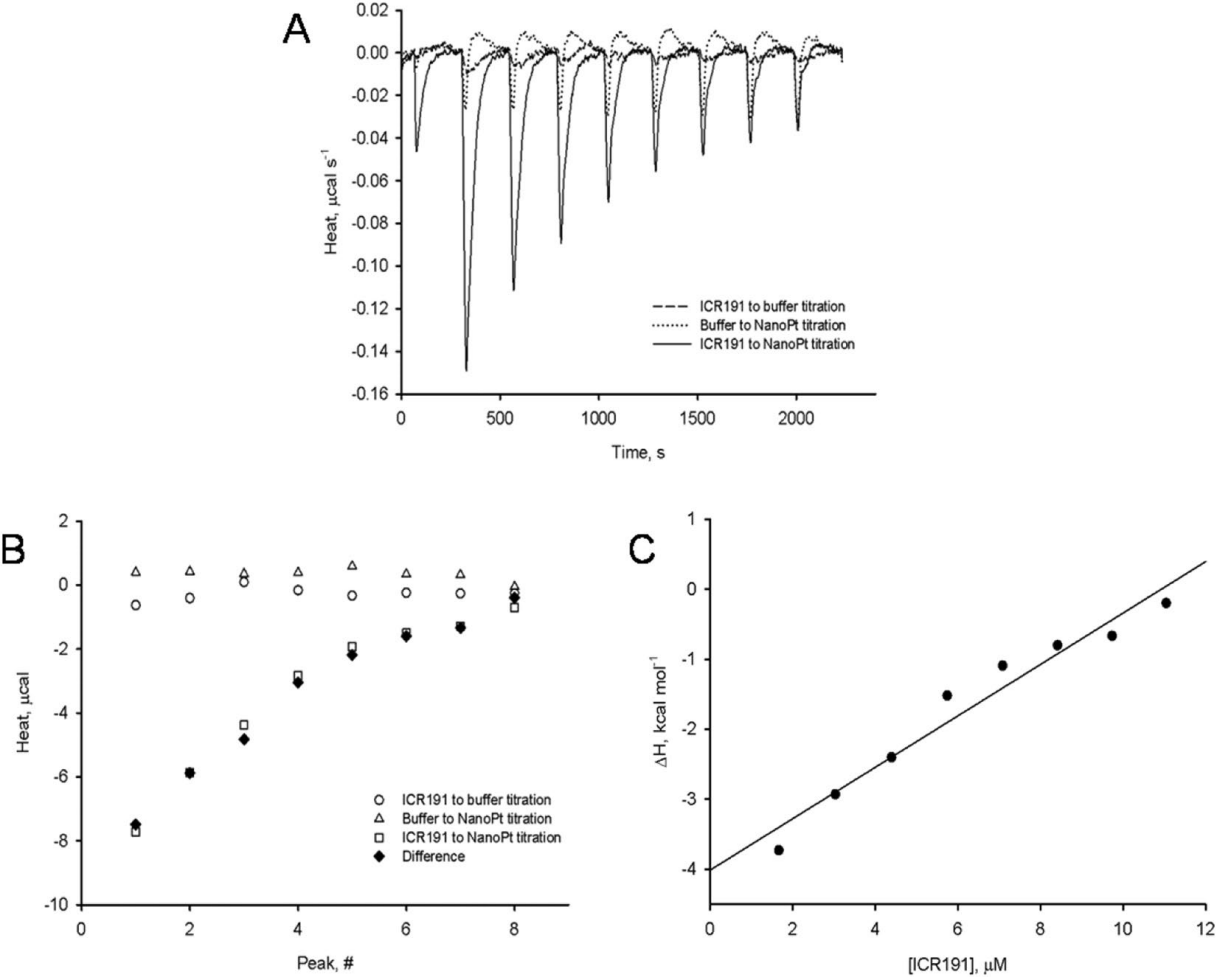
Determination of enthalpy values for PtNPs-ICR-191 interactions: (**A**) thermograms presenting microcalorimetric titrations of PtNPs with ICR-191 (solid line), buffer with ICR-191 (dashed line) and PtNPs with buffer (dotted line), shown as heat released in time and (**B**) thermal effects of titrations of PtNPs with ICR-191 (squares), buffer with ICR-191 (circles) and PtNPs with buffer (triangles). Diamonds represent difference between the heat of PtNPs titration with ICR-191 and sum of heats obtained for control titrations (buffer-ICR-191, PtNPs-buffer), and (**C**) heat of ICR-191-PtNPs interactions (corrected for background thermal effects as described above), calculated as kcal mol^−1^ of injected ICR-191. The enthalpy change (***ΔH***) of ICR-191-PtNPs interactions, calculated by the linear regression (r^2^ = 0.95) of experimental points to [ICR-191] tending to zero, is equal to −4.01 ± 0.23 (±SE) kcal mol^−1^.

To investigate the thermal effects of PtNPs and ICR-191 mixed aggregates formulation, we used Isothermal Titration Calorimetry. Thermograms presenting titrations of ICR-191 with buffer, buffer with PtNPs, and ICR-191 with PtNPs are shown in Fig. 3A. The final thermal effect of PtNPs–ICR-191 interactions was calculated after considering heats of dilution processes of every component in the experiment (Fig. 3B). The heats measured for control samples were subtracted from the heat of PtNPs titration with ICR-191 and the corrected heat is presented on a plot (Fig. 3C). We determined enthalpy changes value for PtNPs–ICR-191 interactions (*ΔH* = −4.01 ± 0.23 kcal·mol^−1^ of injected titrant), calculated by the linear regression (r^2^ = 0.95) of experimental points for ICR-191 concentration tending to zero. Obtained data indicate that aggregates nanoformulation is a result of the spontaneous, exothermic reaction. A comparable negative enthalpy change (*ΔH* = −8.48 ± 0.17 kcal· mol^−1^ of injected titrant) was also observed in a study using direct interaction of acridine mutagen with fullerene C_60_^22^.

There are reports in the literature that suggest that nanostructures such as gold nanoparticles and carbon nanotubes are able to covalently interact with anticancer drugs and efficiently transport them to target cells^3,11,28,29^. On the other hand, some groups have found that nanoparticles, such as fullerene C_60_ and other metal-based nanostructures, can be used as a delivery platform without interacting directly and covalently with the selected active compounds, and in consequence diminishing their biological properties^2,3,22,30,31^. To evaluate the impact of such drug-nanoparticles interactions on drugs biological activity, the bacteriological mutagenicity assay based on *S. typhimurium* TA98 strain can be utilized.

**Figure 4:**
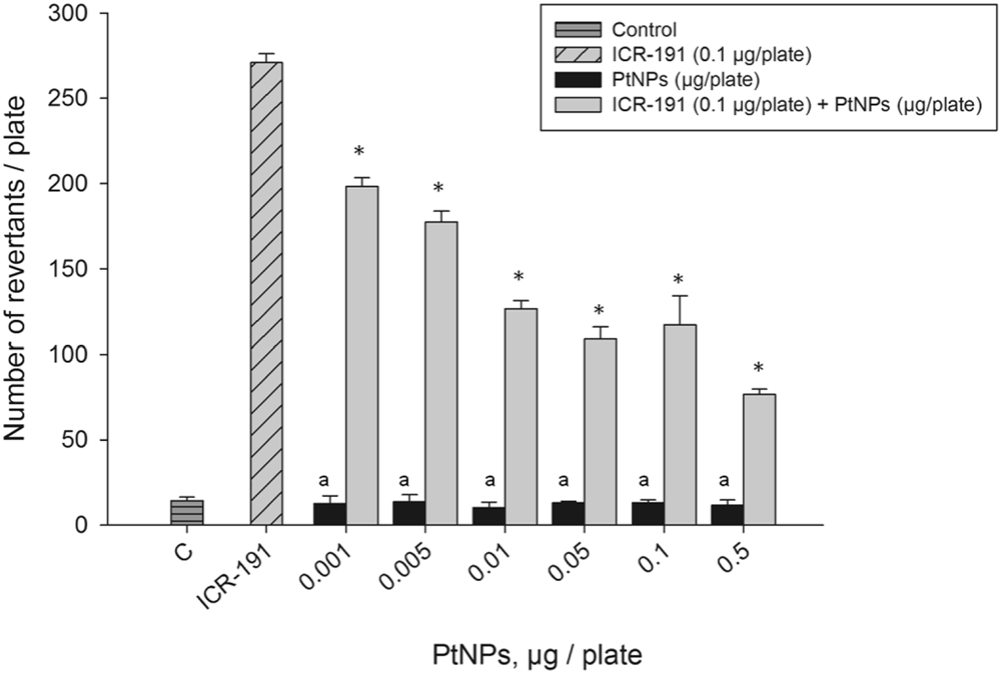
Influence of the platinum nanoparticles (PtNPs) on ICR-191 mutagenic activity in *S. typhimurium* TA98 mutagenicity test. Results are reported as mean number of revertants ± SD. *Values significantly different from the mutagenic level of ICR-191 alone (p < α, α = 0.05); a – no significant difference from the negative control (p > α, α = 0.05).

A significant reduction of the biological activity of ICR-191 was previously observed by our research group, with the use of the Ames test, when reacting this compound with increasing concentrations of fullerene C_60_ nanoparticles^22^. We observed a similar pattern in this study, when the bacteria were treated with 0.1 µg/plate of highly mutagenic ICR-191 associated with PtNPs at concentrations ranging from 0.001 to 0.5 µg/plate (Fig. 4). The reduction of ICR-191 biological activity by PtNPs particles might be interpreted as a temporary protective mechanism to the bacteria, triggered by direct interactions between nanoparticles and mutagen molecules. The highest protection effect was observed for the highest PtNPs concentration (0.5 µg/plate). The sequestration of ICR-191 molecules in heteroaggregates with PtNPs might decrease free mutagen concentration, which reduces its availability for the bacterial cells. The PtNPs alone did not exhibit mutagenic potential towards the bacteria in a tested range of concentrations (Fig. 4). Lack of a nanoplatinum mutagenicity towards *S. typhimurium* TA98 was also observed by Maenosono *et al*.^29^, while they described a light mutagenicity Fe-Pt nanoparticles towards *S. typhimurium* TA100^17^.

**Figure 5:**
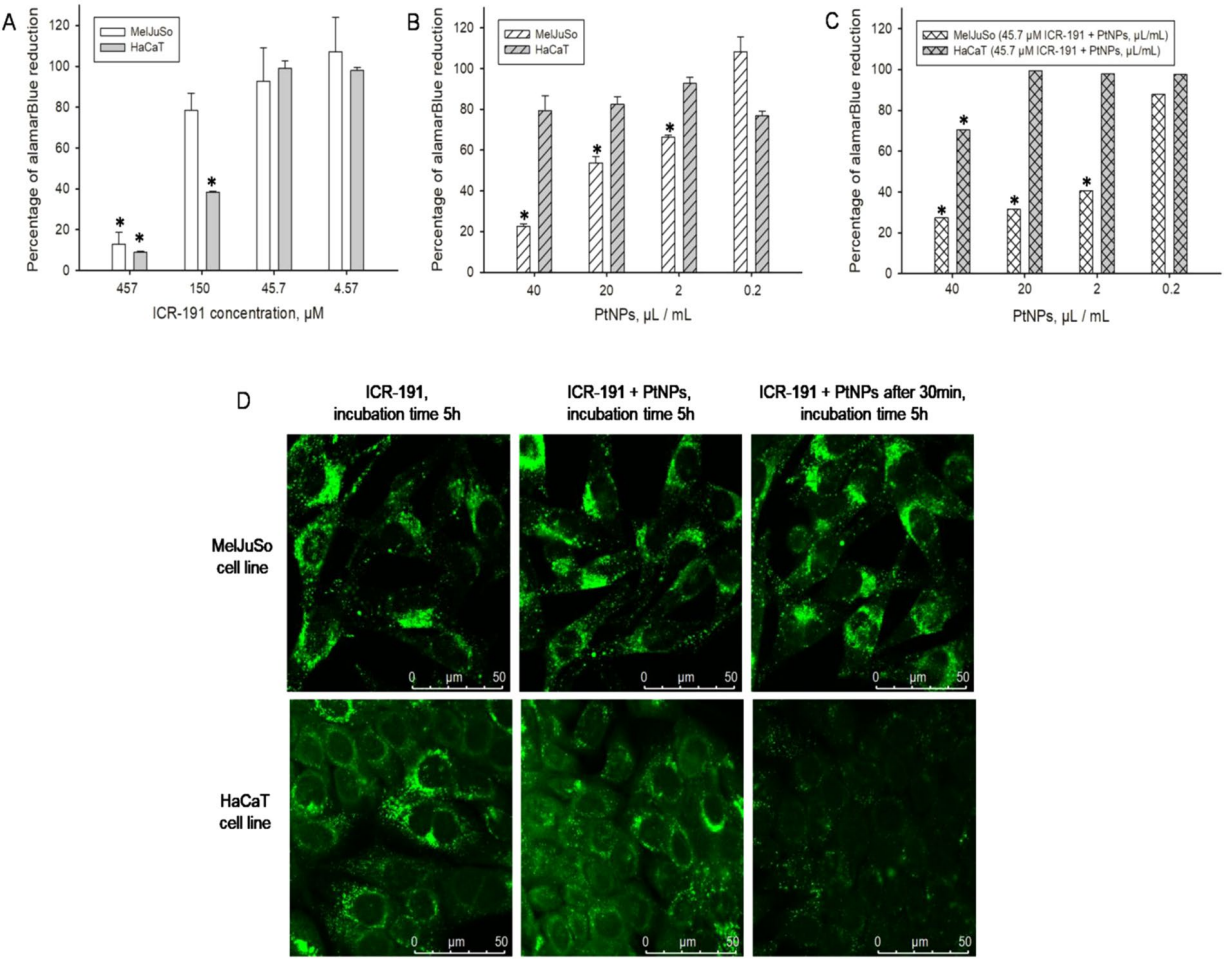
Influence of the platinum nanoparticles (PtNPs) on ICR-191 mutagenic activity in tested eukaryotic cell lines: (**A**) comparison of the cell viability of HaCaT and MelJuSo cell lines incubated for 72 h in a presence of ICR-191 (4.57–450 µM), based on alamarBlue assay. Data are expressed as the mean ± standard deviation and (**B**) comparison of the cell viability of HaCaT and MelJuSo cell lines incubated for 72 h in a presence of PtNPs (0.2–40 µL/mL), based on alamarBlue assay. Data are expressed as the mean ± standard deviation and (**C**) comparison of the cell viability of HaCaT and MelJuSo cell lines incubated for 72 h in a mixture of ICR-191 (45.7 µM) and PtNPs (0.2–40 µL/mL), based on alamarBlue assay. Data are expressed as the mean ± standard deviation, and (**D**) confocal microscopy live analysis of the impact of platinum nanoparticles (PtNPs) on ICR-191 fluorescence in the HaCaT and MelJuSo cell lines. Cells were treated with 45.7 µM ICR-191; treated with 45.7 µM ICR-191 and 3 ng/mL PtNPs mixture, or preincubated with 3 ng/mL PtNPs and subsequently treated with 45.7 µM ICR-191 (PtNPs preincubation). Time of incubation in the presence of ICR-191 indicated above particular panels. *Values significantly different from the amount of alamarBlue reduced by untreated control cells (p < α, α = 0.05).

During this study, we also aimed to evaluate and visualize the possible effects of nanoplatinum in the ICR-191 biological activity towards eukaryotic cell lines: non-cancerous HaCaT and cancerous MelJuSo. To address this problem we used the cells’ viability assay based on alamarBlue. During this test, only metabolically active cells are able to convert the reagent into a colorimetric indicator. In the first approach, to evaluate mutagenic potential of ICR-191 alone, cells were incubated for 72 hours in its broad range of concentrations (4.57–457 µM). The percentage calculated of alamarBlue reduction was then plotted against mutagen concentration and presented in Fig. 5A. Before the main experiment we carried out an additional analysis determining the ICR-191 half-maximal inhibitory concentration (IC_50_) for both tested cell lines (obtained values equal 0.22 mM for HaCaT and 0.28 mM for MelJuSo). We noticed that the increasing ICR-191 concentration corresponds to the decreasing cellular metabolic activity. In addition, ICR-191 has stronger impact on non-cancerous HaCaT cells than on cancerous MelJuSo cells. In opposition to tested mutagen, platinum nanoparticles after 72 hours incubation (checked concentrations range: 0.2–40 µL/mL) have no influence on HaCaT cells, but do reduce MelJuSo cells proliferative ability (Fig. 5B). That observation was additionally confirmed by nanoplatinum IC_50_ determination. To trigger the half-maximal inhibitory effect in MelJuSo cells 23 µL/mL of PtNPs is needed, while for HaCaT cells required concentrations is 102 µL/mL. Furthermore, MelJuSo cells incubated with mixtures containing ICR-191 (45.7 µM) and changing amount of PtNPs (0.2–40 µL/mL) also showed reduced viability, comparing to HaCaT cells (Fig. 5C). In that case, nanoplatinum IC_50_ concentration determined for HaCaT cells was one hundred times greater than for MelJuSo cells (1.65 µL/mL and 166.9 µL/mL respectively). Taking it into consideration, it might be presumed that PtNPs have a dose dependent toxic activity against MelJuSo cells, and what is even more interesting, combination of mutagen with nanoparticles leads to a decrease in the toxicity against non-neoplastic cells, while maintaining the level of this activity against cancerous ones.

In next step, applied confocal microscopy technique allowed us to determine the impact of PtNPs alone and mixed with ICR-191 on single cells in a real-time mode. Both cell lines were treated with PtNPs, ICR-191, or their mixtures. Emitted fluorescence was measured over time after 5 hours incubation, and is presented in Fig. 5D. The first vertical panel shows images of cells after treatment with ICR-191 (45.7 µM). The ICR-191 fluorescence was identical in both cell lines. The middle panel shows cells treated with a mixture consisting of PtNPs and ICR-191 (3 ng/mL and 45.7 µM, respectively). The last panel presents the fluorescence of the cells preincubated with PtNPs (3 ng/mL) for 30 minutes, and then stained with ICR-191 (45.7 µM). These images showed visible reduction of ICR-191 signal in HaCaT cells, which were previously incubated with PtNPs, meanwhile fluorescence intensity in MelJuSo cells did not change. PtNPs influence on ICR-191 biological activity stands in agreement with data obtained in the spectrofluorimetric analysis and it correlates with the toxicity assays results. This phenomenon could be interpreted as a protective mechanism of nanoplatinum against mutagen activity toward eukaryotic cells, connected with the heteroaggregates formation. We suspect that ICR-191 molecules involved in aggregates formation with nanoparticles are temporarily disabled from their mutagenic and toxic activity. Interestingly, this quenching effect is observed only in non-cancerous cells, while the fluorescence intensity of ICR-191 in cancerous MelJuSo cells is maintained.

It should be underline that platinum nanoparticles are already known for their broad anticancer activities. The several findings indicate PtNPs uptake to target cells by diffusion or endocytosis and then their aggregation inside cells^14–17,32,33^. PtNPs have a strong affinity to the mitochondria and can overcome blood-brain barrier^14,15^. In addition, PtNPs abilities to activate p53 and kill HeLa cells^17^, to induce apoptosis in brain cells^15^, to inhibit U87, U118 and U251 glioblastoma tumor cells proliferation^2,12,17^, to deform human red blood cells^21,32^, as well as to enhance strongly lethal damage in DNA in the hadron therapy^5^ are widely described. In summary, the present findings in association with data published in the literature, lead us to conclude that PtNPs are excellent candidates for use as putative transporting platform and co-treating factors with other active substances. Notwithstanding, to fully understand the mechanism of interaction between mutagen and nanoparticles, it is necessary that further research to be conducted.

**Figure 6:**
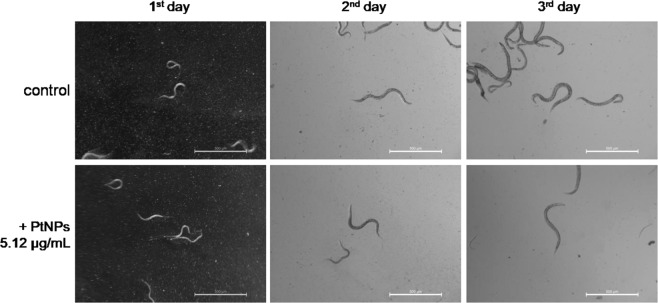
Survival rate of *Caenorhabditis elegans* untreated and treated with PtNPs. Tested nematodes were treated with PtNPs in a broad range of concentrations (0.04–5.12 µg/mL). Pictures were taken after one, two and three days of incubation.

In the last approach, we estimated the potential toxicity of newly synthesized PtNPs towards multicellular model animal *C. elegans*, an optimized tool for diverse nanoparticles characterization^34,35^. Any significant changes in survival rate were monitored after 24 h treatment. Our results demonstrated that PtNPs were not toxic towards the nematode analysed Fig. S4 (Supporting Information) in the tested range of concentrations (0.04–5.12 µg/mL). Moreover, PtNPs did not impair the life cycle of worms (Fig. 6). In a similar manner to the control group, animals maintained in the medium supplemented with 5.12 µg/mL developed into their adult form and laid eggs. Our results confirmed the non-toxicity of PtNPs towards *C. elegans*, as described so far in the literature^19,36–38^.

## Conclusions

While applying nanodelivery strategy for biologically active agents, the main advantages of the use of nanoparticles as transporting platforms relate to their ability to efficiently target tumor tissue and release drugs molecules in a controlled way. This study verified that PtNPs are able to interact directly with ICR-191, a model biologically active compound, while being synthesized in a cost and time effective manner. Moreover, the biological assays’ results confirmed that these kind of metal-based nanoparticles should be considered as potential delivery platforms for biologically active substances. However, a more detailed analysis, involving a large group of drugs, is likely necessary.

## Materials and Methods

### Materials and preparation procedures

Model acridine mutagen ICR-191 was purchased from Sigma Aldrich Chemical Company. Its stock solution (concentration 10^−3^ M) was obtained by dissolving the weight amount in distilled water. Concentrations of ICR-191 solutions was determined spectrophotometrically, using molar absorption coefficients ε_421.5_ = 7.5624 × 10^3^ M^−1^ cm^−1^. *Salmonella typhimurium* TA98 strain used in the mutagenicity Ames test was purchased from Xenometrics AG, Allschwil, Switzerland. Ampicillin, biotin and histidine used in the Ames test were purchased from Sigma Aldrich Chemical Company. Nutrient Broth media were bought in EMAPOL, Gdańsk, Poland. In toxicity assays, the human keratinocyte cell line (HaCaT) and the human melanoma cell line (MelJuSo) were employed. HaCat cells were obtained from the Department of Microbiology, Tumor and Cell Biology, Karolinska Institute (Stockholm, Sweden), while the MelJuSo cells were from Department of Medicinal Microbiology, Leiden University Medical Center (Leiden, The Netherlands). The Dulbecco’s modified Eagle’s medium (DMEM), bovine serum, L-glutamine, glucose, penicillin and streptomycin used in the cell lines experiments were purchased from Sigma- Aldrich. *Caenorhabditis elegans* wild-type strain Bristol N2 was provided by the Caenorhabditis Genetic Center (CGC), which is funded by NIH Office of Research Infrastructure Programs (P40 OD010440).

### Construction of the Continuous Flow Reactor/Platinum Nanoparticles Synthesis

**Figure 7:**
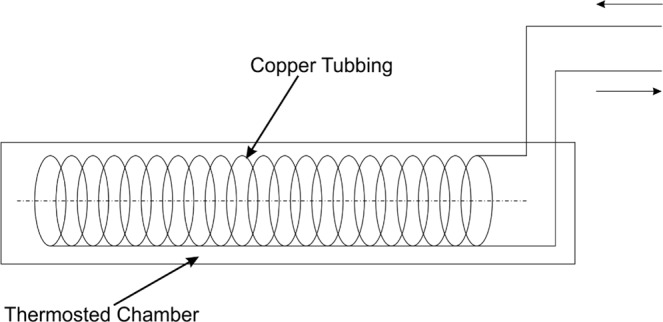
Scheme of the platinum nanoparticles synthesis reactor.

The flow reactor was made with the use of a 5-meter copper capillary tubing (external diameter 2.5 mm, wall thickness 0.6 mm) that was coiled leaving straight 25 cm ends on both sides. The coiling diameter was 60 mm. One end of the capillary was fitted with a 20 mL syringe, and the second end with collection reservoir. The coiled part of the capillary was placed in a thermostat-controlled water bath (Fig. 7). Platinum nanoparticles were synthesized using a buffer that consisted of 80 mM l-ascorbic acid (Sigma-Aldrich) and 16 g/L polyvinylpyrrolidone (Sigma-Aldrich). The pH of the buffer was set to either 5 or 7 depending on the performed experiment. Before the synthesis, the buffer was mixed in 1:1 ratio with 32 mM potassium hexachloroplatinate (Merck). The obtained yellowish solution was fed with a syringe through the flow reactor with the speed of 1 mL per minute. The reaction temperature was set to 90 °C. The black solution obtained after passing through the reactor was collected and filtered through a sterile 0.22 µm mixed cellulose ester syringe filter (VWR International). The final nanoparticles solution (at concentration 25.6 mg/L) was kept at room temperature for further testing, for no longer than two weeks prior to the experiments.

### Transmission Electron Microscopy – TEM

PtNPs samples were placed on grids (Sigma-Aldrich, St Louis, MO, USA) coated with a 2% collodion solution (Sigma-Aldrich). Afterwards, samples absorbed to the surface were examined using a Philips CM100 electron microscope at 80 kV (FEI Company, Eindhoven, Netherlands).

### Dynamic Light Scattering – DLS

Examination of the aggregates size distributions for PtNPs alone (0.16 µg/mL) and PtNPs in mixture with ICR-191 (final concentrations respectively: 0.15 µg/mL and 0.12 mM) were conducted by DLS on the Zetasizer Nano ZS (Malvern, Worcestershire, UK) by measuring the intensity of the scattered light. The analyzed mixtures were placed into polystyrene cuvettes. All measurements were performed at 25 °C with a He-Ne laser (633 nm, 4 mW), at a 173° scattering angle, as was previously described in Borowik *et al*.^22^. Results were evaluated using the Smoluchowski approximation, which is known to be rigorously valid only for spherical-like particles. Obtained data are shown as a size distribution [nm] of light scattering particles (in accordance to their hydrodynamic diameters) by intensity [%].

### Scanning electron microscopy-energy dispersive X-ray spectrometry – SEM-EDS

Qualitative standardless chemical analysis of PtNPs was performer using scanning electron microscope (Nova NanoSEM 200, FEI) coupled with a Genesis XM X-ray microanalysis system (EDAX) featuring the Sapphire Si(Li) energy dispersive X-ray (EDX) detector. The measurements and morphology observation were done in low vacuum conditions using Helix detector.

### Fluorescence Spectroscopy

The intensity of fluorescence spectra was determined for ICR-191 alone (Supporting Information, Fig. S3) and for its two mixtures with platinum nanoparticles. In the first approach, ICR-191 fluorescence emission spectrum (excitation wavelength = 340 nm, emission wavelengths = 400–650 nm) was measured for PtNPs (primary concentration: 32 µg/mL) titrated with increasing amounts of ICR-191 (concentration range: 13–123 µM). In the second experiment, ICR-191 (primary concentration: 124 µM) was titrated with increasing amount of PtNPs (concentration range: 3.2–32 µg/mL). All samples were measured in quartz cuvettes at 25 °C, using FP-8500 Spectrofluorometer (Jasco, Easton, Maryland, USA). Obtained data are expressed as the mean relative fluorescence unit (RFU).

### Isothermal Titration Calorimetry – ITC

The microcalorimetric measurements were performed at 25 °C, using AutoITC isothermal titration calorimeter (MicroCal Inc. GE Healthcare, Northampton, USA), as was described before^16^. ICR-191 and PtNPs used in this procedure were dissolved in synthesis buffer, pH = 5. The volume of both sample and reference cells (which contains synthesis buffer) was 1.4491 mL. Experiment consisted of multiple injections of 10 µL ICR-191 portions (initial concentration 0.200471 mM) to the sample cell containing PtNPs solution (initial concentration 0.1312 mM), followed by the measurements of the heat of the process as a function of time. Two additional background titrations were performed, using ICR-191 solution with synthesis buffer and synthesis buffer as a titrant with PtNPs. Each injection lasted 20 s. In order to achieve a homogeneous mixing in the cell, the stirrer speed was kept constant at 300 rpm. Registered results of background titrations were subtracted from final experimental results to account for the heat of dilution. Obtained data were processed with Origin 7 from MicroCal, and finally expressed as heat/mole of added mutagen (kcal mol^−1^).

### Ames Mutagenicity Test

The bacterial mutagenicity test (called Ames test) was performed with *Salmonella typhimurium* TA98 strain, without metabolic activation, in accordance with the procedure described by Mortelmans and Zeiger^39^ with further modifications described in Golunski *et al*.^40^. The mixture of 100 µL of overnight culture of *S. typhimurium* TA98 (corresponding to 1 × 10^8^ colony forming units), 50 µL of 3% NaCl, and 100 µL of tested compounds dissolved in water (sterile water was used for the negative control) was incubated for 4 h in darkness at 37 °C and 220 rpm. Afterwards, the mixture was centrifuged, bacterial pellet washed with 0.75% NaCl, and resuspended in 300 µL of 0.75% NaCl solution containing 0.1 µmol histidine and 0.1 µmol biotin. Finally, the cell suspension was spread on glucose minimal (GM) plate. Number of revertant colonies were calculated after cultivation for 48 hours at 37 °C in darkness. All experiments were done in triplicate. Determination of ICR-191 mutagenic activity in a broad concentration range allows for the optimal concentration of the mutagen (0.1 µg/plate) for subsequent analysis in the mixtures with PtNPs was selected in a preliminary experiment in which ICR-191 mutagenic activity in a broad range of concentrations was assessed. Possible toxicity towards bacteria was determined by observation of the auxotrophic background (background lawn). PtNPs alone were not mutagenic towards bacteria in tested range of concentrations (0.001–0.5 µg/plate).

### Cell culture, and Life Analysis of Cell Lines by Confocal Microscopy

Cell lines HaCaT and MelJuSo were cultured in Dulbecco’s modified Eagle’s medium (DMEM) containing 4500 mg/L glucose, supplemented with 10% fetal bovine serum, 4 mM L-glutamine, 100 units/mL penicillin and 100 mg/mL streptomycin. Cultures were maintained in a humidified atmosphere containing 5% CO_2_ at 37 °C. HaCaT (10^5^) as well as MelJuSo (10^5^) cells were seeded on a 35 mm glass bottom plate (WillCo Wells) and allowed to grow overnight. Afterwards, cells were washed three times with DMEM medium devoid of FBS and antibiotics. Then, cells were incubated for 5 h either with ICR-191 (45.7 µM), PtNPs (3 ng/mL), or the mixture of ICR-191 and PtNPs diluted in the FBS- and antibiotics-free medium. In order to investigate the cellular localization of ICR-191 after preincubation with PtNPs, cells were treated with PtNPs alone and incubated for 1.5 h at 37 °C, in a humidified atmosphere containing 5% CO_2_, before the addition of ICR-191. As acridine mutagen is an example of fluorescent compound, there was a possibility to detect the intracellular localization by confocal microscopy using Leica SP8 confocal laser scanning microscope system (Leica, Wetzlar, Germany), equipped with an incubation chamber for the live analysis. The excitation wavelength was set to 340 nm and emission was detected at 496 nm. Observation was conducted at 37 °C, in a humidified atmosphere containing 5% CO_2_, as was previously described in Golunski *et al*.^41^.

### Cell Viability Assay

HaCaT and MelJuSo cells were seed on a 96-well plate (2 × 10^4^/well) and incubated in humidified atmosphere containing 5% CO_2_, at 37 °C, overnight. Then, different dilutions of ICR-191, PtNPs, or mixture of PtNPs and ICR-191 (90 µl/well) were added to the tested cell cultures in three replicates and incubated for 72 h. The additional control wells consisted only of untreated cells with the media. Subsequently, 10 µl of alamarBlue (Bio-Rad) was added into each well and further incubated for the next 4 h, in humidified atmosphere containing 5% CO_2_, at 37 °C. Finally, absorbance was measured at wavelengths of 570 nm and 600 nm. Pure media was used as blank. Percentage of the alamarBlue reduction was calculated as the difference between treated and control cells, according to the protocol provided by the manufacturer. Half-maximal inhibitory concentrations (IC_50_) were determined according to the instructions provided by the manufacturer. Obtained data are presented as the mean ± standard deviation.

### Toxicity Testing on *C. elegans*

Nematodes were cultured and synchronised according to published protocol^34^. Toxicity of PtNPs (concentration range: 0.04–5.12 µg/mL) was tested towards synchronised *C. elegans* L4 larvae cultured in S complete medium supplemented with *Escherichia coli* strain OP50 (1 × 10^8^ CFU/mL). After 24-hour incubation the number of living worms was established by worms counting under the stereomicroscope (Leica MZ10F). All cultures and tests were done at 25 °C.

### Statistical analysis

The results from mutagenicity assays were evaluated statistically with Statistica 12 (StatSoft) software. One-way variance analysis (ANOVA) followed by the post-hoc RIR Tukey’s test was applied. Significance level was established at α = 0.05.

## Supplementary information

Supplementary Information
