# Supplementary material for: Interactions of newly synthesized platinum nanoparticles with ICR-191 and their potential application
Source: Sci Rep. 2019 Mar 21;9:4987. doi: 10.1038/s41598-019-41092-6 (PMC6428851; doi:10.1038/s41598-019-41092-6)
Supplement: Supplementary file 1 — Supplementary Information [file 41598_2019_41092_MOESM1_ESM.docx]

Electronic Supplementary Material

| **Interactions of newly synthesized platinum nanoparticles with ICR-191 and their potential application** |
| --- |
| Agnieszka Borowik^1^, Rafal Banasiuk^2^, Natalia Derewonko^3^, Michal Rychlowski^3^, Marta Krychowiak-Masnicka^2^, Dariusz Wyrzykowski^4^, Anna Woziwodzka^1^, Magdalena Ziabka^5^, Aleksandra Krolicka^2^ (**🖂**), and Jacek Piosik^1^ (**🖂**)  *^1^ University of Gdansk, Intercollegiate Faculty of Biotechnology UG and MUG, Laboratory of Biophysics, Abrahama 58,* *Gdansk 80-307, Poland*  *^2^ University of Gdansk, Intercollegiate Faculty of Biotechnology UG and MUG, Laboratory of Biologically Active Compounds,* *Abrahama 58,* *Gdansk 80-307, Poland*  *^3^ University of Gdansk, Intercollegiate Faculty of Biotechnology UG and MUG, Laboratory of Virus Molecular Biology, Abrahama 58,* *Gdansk 80-307, Poland*  *^4^ University of Gdansk, Faculty of Chemistry,* *Wita Stwosza 63,* *Gdansk 80-308, Poland*  *^5^AGH University of Science and Technology, Faculty of Materials Science and Ceramics, Department of Ceramics and Refractories, Krakow 30-059, Poland*  Supporting information to DOI 10.1007/s12274-****-****-* (automatically inserted by the publisher) |

**Methods**

**UV-vis Spectrophotometric Measurements**

Light absorption spectra were measured in a wide wavelength range 300-800 nm with 0.5 nm intervals, using Beckman’s DU 650 spectrophotometer with temperature stabilized by Polystat thermostat constant circulator (25 ± 0.1° C). Experiments were done in quartz cuvettes (1 cm light path) containing 2 mL 0.2 M sodium-phosphate buffer, pH 6.8, which was titrated with platinum nanoparticles (PtNPts) and ICR-191.

**Fluorescence Spectroscopy**

The intensity of fluorescence spectra was determined for ICR-191 solution, final concentration: 124 µM. The samples were measured in quartz cuvettes at 25°C, using FP-8500 Spectrofluorometer (Jasco, Easton, Maryland, USA). Obtained data are expressed as the mean relative fluorescence unit (RFU).

**Toxicity testing on** ***Caenorhabditis elegans***

*C. elegans* were cultured and synchronised according to published protocol [1]. Toxicity of PtNPs (concentration range: 0.04–5.12 µg/mL) was tested towards synchronised C. elegans L4 larvae cultured in S complete medium supplemented with Escherichia coli strain OP50 (1 × 108 CFU/mL). After 24-hour incubation the number of living worms was established by worms counting under the stereomicroscope (Leica MZ10F). All cultures and tests were done at 25°C.

**Results**

**UV-vis spectrophotometric measurements**

To verify possible interactions between ICR-191 and platinum nanoparticles we performed two spectrophotometric experiments. Results of the first one - the absorption spectra of ICR-191 (initial concentration 57.26 µM) titrated with PtNPs in a broad range of concentrations (0.05 – 0.768 µg/mL) in 0.2 M sodium-phosphate buffer (pH=6.8) are presented in Figure S1a. Registered data were then normalized to ICR-191 concentration and presented in a form of molar extinction spectra in Fig. S1B. Unfortunately, PtNPs tend to form homoaggregates in tested range of concentrations which intensively scatter the light. What is more, spectra of PtNPs and ICR-191 (published by Gołuński et al.[2]) are overlapping, which prevents its further thermodynamical analysis.


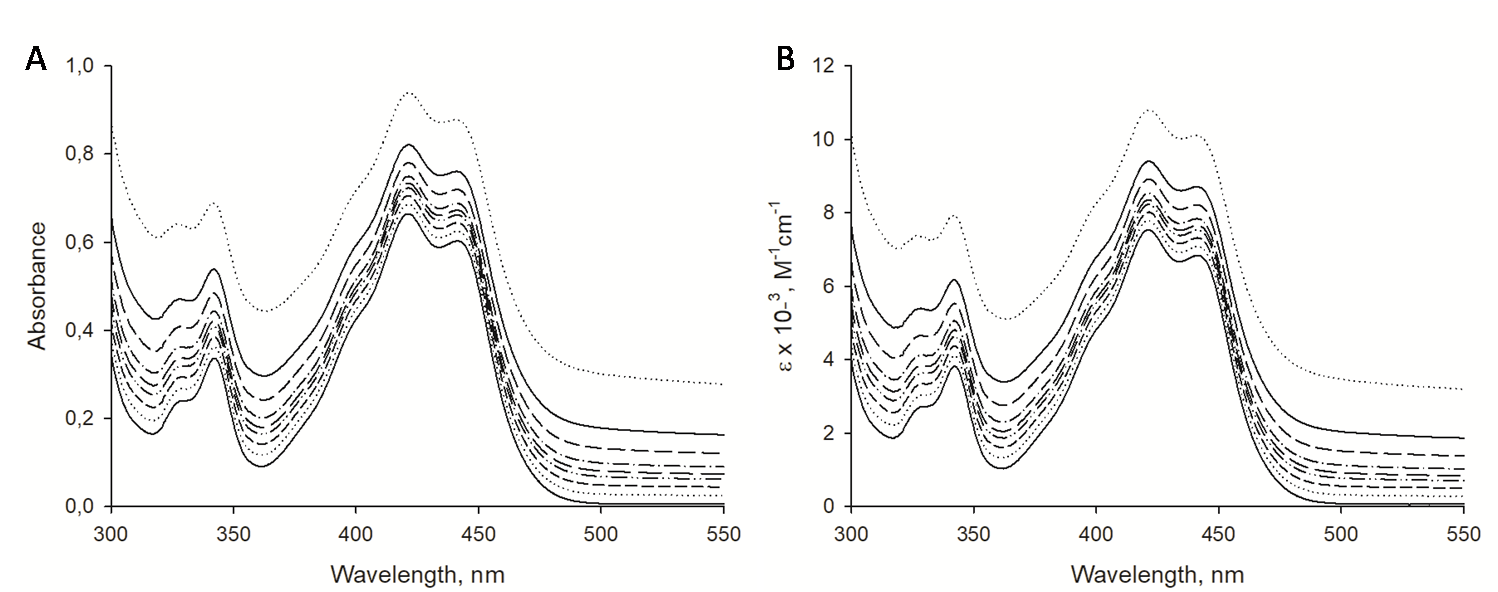


**Figure S1** Spectrophotometric analysis of ICR-191 - platinum nanoparticles aggregation: a) absorbance spectra of ICR-191 (initial concentration 57.26 µM) titrated with PtNPs (0.05 – 0.768 µg/mL concentration range) in 0.2 M sodium-phosphate buffer (pH=6.8) and b) data were normalized to ICR-191 concentration and presented in the form of molar extinction coefficient.

In the second experiment, PtNPs (initial concentration 7.68 µg/mL) was titrated with ICR-191 (concentration range 8.54-80.63 µM). To minimize impact of strong PtNPs homoaggregation, absorbance spectra of PtNPs titrated with distilled water (added in the same volumes as tested mutagen) were subtracted from the newly registered data. Resulting spectra are shown in Figure S2a. Next, obtained data were normalized to ICR-191 concentration and presented as a molar extinction coefficient spectra in Figure S2b. Slight bato- and hypochromic shifts are observed, indicating the existence of direct interactions between PtNPs and ICR-191.


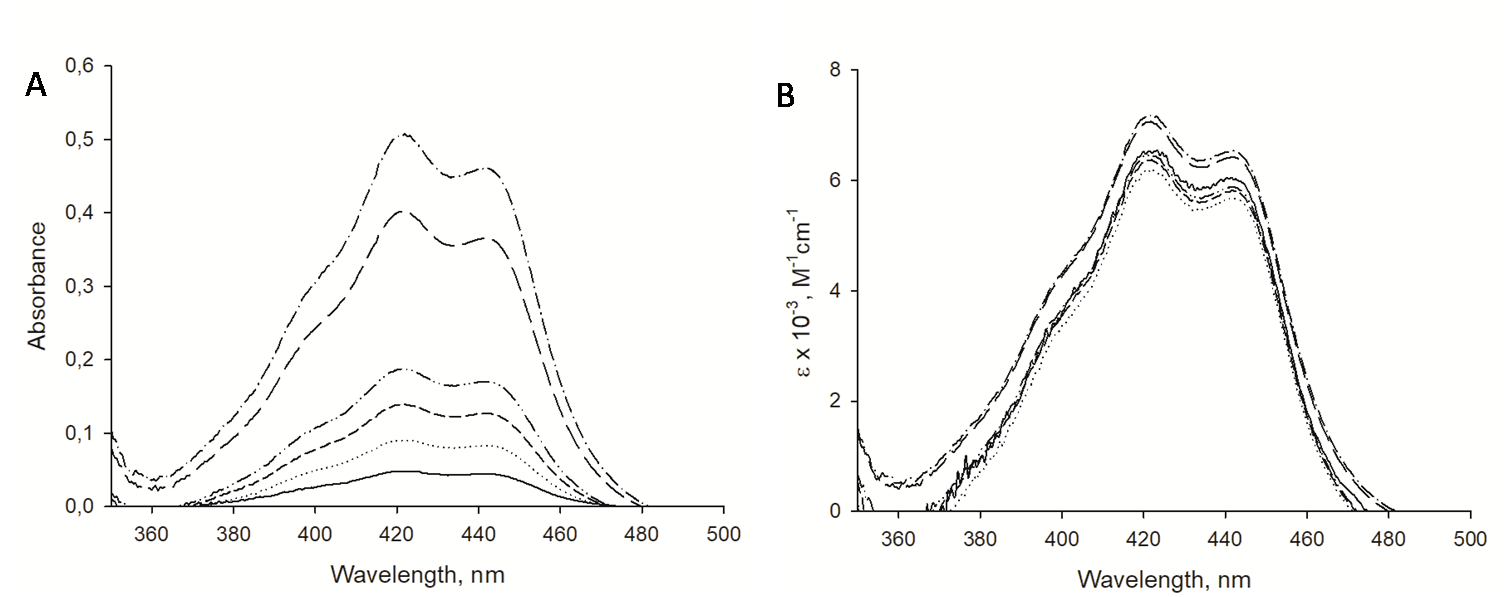


**Figure S2** Spectrophotometric titrations of platinum nanoparticles (PtNPs) with ICR-191 reduced by PtNPs absorbance: a) absorbance spectra of PtNPs (initial concentration 7.68 µg/mL) titrated with ICR-191 (concentration range 8.54-80.63 µM). To minimize effect of PtNPs homoaggregation, absorbance spectra of PtNPs titrated with distilled water (added in the same volumes as tested mutagen) were subtracted from the newly registered data and b) data were normalized to ICR-191 concentration and presented in a form of molar extinction coefficient.

**Mutagen fluorescence spectroscopy**

The intensity of fluorescence light emitted by ICR-191 (final concentration 124 µM) was measured with excitation wavelength = 340nm and emission wavelength = 400-600nm. The registered data are presented in Figure S3 as the mean relative fluorescence unit (RFU). The maximum fluorescence point was found at 496 nm.


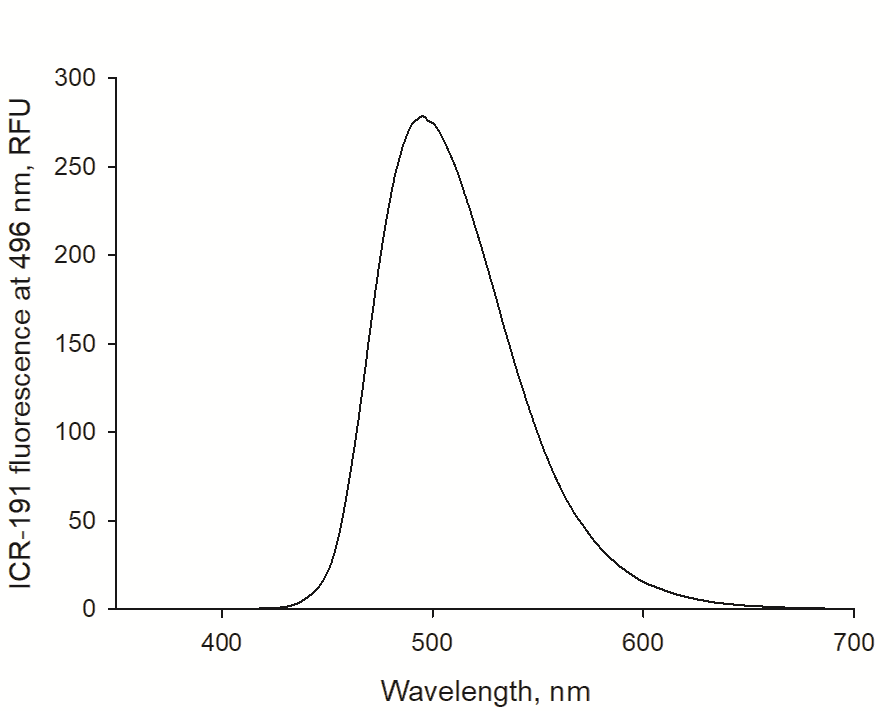


**Figure S3** Registered ICR-191 emission spectrum. ICR-191 fluorescence emission spectrum (Ex = 340 nm, Em = 400–650 nm) measured for ICR-191 solution (final concentration: 124 µM). Obtained data are expressed as the mean relative fluorescence unit (RFU).

**Survival rate in *Caenorhabditis elegans***

In the last experiment, we estimated the potential toxicity of newly synthesized PtNPs towards multicellular model animal *C. elegans*. Any significant changes in survival rate were monitored after 24h treatment. Our results demonstrated that PtNPs were not toxic towards the nematode analysed in the broad range of concentrations (0.04–5.12 µg/mL). Moreover, PtNPs did not impair the life cycle of worms – they developed into their adult form and laid eggs (Figure S4).


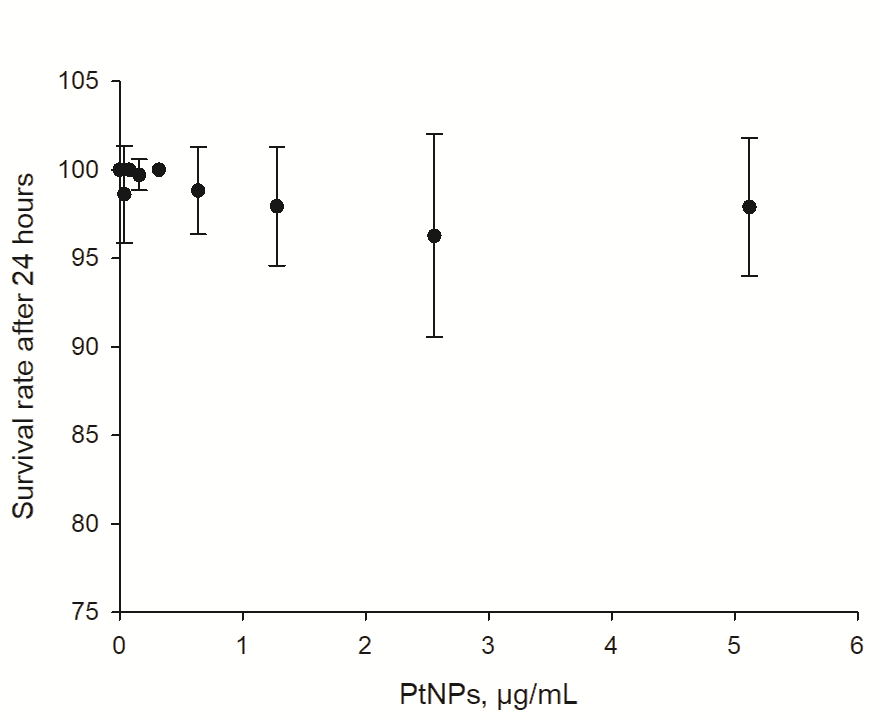


**Figure S4** Survival rate of *Caenorhabditis elegans* treated with platinum nanoparticles (PtNPs). Tested nematodes, treated with PtNPs in a broad range of concentrations (0.04–5.12 µg/mL), did not show any changes in survival rate. Obtained data are presented as the mean ± standard deviation.

**References**

1. Stiernagle, T. Maintenance of C. elegans. WormBook **2006**, 1–11. doi:10.1895/wormbook.1.101.1.
2. Gołuński, G.; Woziwodzka, A.; Iermak, I.; Rychłowski, M.; Piosik J. Modulation of acridine mutagen ICR191 intercalation to DNA by methylxanthines - Analysis with mathematical models. *Bioorg. Med. Chem.* **2013**, *21*, 3280–3289.
